# Etiology of Puerperal Eclampsia—Gerdes

**Published:** 1893-06

**Authors:** J. O. Lewright

**Affiliations:** Austin


For Daniel's Texas Medical Journal:

### ETIOLOGY OF PUERPERAL ECLAMPSIA—GERDES.

J. O. LEWRIGHT, M. D., AUSTIN.

BELOW you will find a short abstract of an article from *Muncheuer Med. Wochenschrift*, 1892, No. 22, which is in line with those treated of in the JOURNAL:

In a case of severe puerperal eclampsia a bacteriological examination was made of the kidneys, the liver, the lungs, and of blood from the aorta. All slides prepared from these organs, took after 24 hours in the incubator. The culture, which is quite accurately described, consisted of short bacilli, which stained when fully developed, and were morphologically similar to the group of bacilli found in chicken cholera, swine plague and rabit septicæmia. In the hanging drop this bacillus shows a lively motion of its own. It is decolorized by the Gram method. Gerdes was also able to demonstrate the bacilli in sections of the organs of the eclamptic. This bacillus has a very deadly effect upon rats and mice. The latter, soon after the subcutaneous injection of 1-10 ccm. of a fifteen-hour old bacillus culture, are seized with vomiting and convulsions, and finally die comatose. In rats, no convulsions, but great listlessness and inertness were observed, and they finally lapsed into complete unconsciousness.

